# Recent Advances in Sample Preparation for Cosmetics and Personal Care Products Analysis

**DOI:** 10.3390/molecules26164900

**Published:** 2021-08-13

**Authors:** Maria Celeiro, Carmen Garcia-Jares, Maria Llompart, Marta Lores

**Affiliations:** 1 CRETUS Institute, Department of Analytical Chemistry, Nutrition and Food Science, Universidade de Santiago de Compostela, E-15782 Santiago de Compostela, Spain; carmen.garcia.jares@usc.es (C.G.-J.); maria.llompart@usc.es (M.L.); 2 Laboratory of Research and Development of Analytical Solutions (LIDSA), Department of Analytical Chemistry, Nutrition and Food Science, Universidade de Santiago de Compostela, E-15782 Santiago de Compostela, Spain; marta.lores@usc.es

**Keywords:** sample preparation, microextraction techniques, miniaturization, cosmetics, personal care products

## Abstract

The use of cosmetics and personal care products is increasing worldwide. Their high matrix complexity, together with the wide range of products currently marketed under different forms imply a challenge for their analysis, most of them requiring a sample pre-treatment step before analysis. Classical sample preparation methodologies involve large amounts of organic solvents as well as multiple steps resulting in large time consumption. Therefore, in recent years, the trends have been moved towards the development of simple, sustainable, and environmentally friendly methodologies in two ways: (i) the miniaturization of conventional procedures allowing a reduction in the consumption of solvents and reagents; and (ii) the development and application of sorbent- and liquid-based microextraction technologies to obtain a high analyte enrichment, avoiding or significantly reducing the use of organic solvents. This review provides an overview of analytical methodology during the last ten years, placing special emphasis on sample preparation to analyse cosmetics and personal care products. The use of liquid–liquid and solid–liquid extraction (LLE, SLE), ultrasound-assisted extraction (UAE), solid-phase extraction (SPE), pressurized liquid extraction (PLE), matrix solid-phase extraction (MSPD), and liquid- and sorbent-based microextraction techniques will be reviewed. The most recent advances and future trends including the development of new materials and green solvents will be also addressed.

## 1. Introduction

The use of cosmetics and personal care products is increasing worldwide, and Europe is a key market for the cosmetics industry. In fact, the European cosmetic market generated more than EUR 120,000 billion in 2019 [1].

Regulation 1223/2009 [2] includes the definition of cosmetics and establishes rules to be complied with by any cosmetic product made placed on the market, to ‘ensure the functioning of the internal market and a high level of protection of human health’. For this purpose, it provides a ‘List of substances prohibited in cosmetic products’ (Annex II) which currently includes more than 1400 chemical compounds. On the other hand, Annex III lists the substances allowed for use as cosmetic ingredients, although most of them present restrictions in terms of maximum permitted concentration depending on the use of the final product or the product type. In addition, different positive lists of substances for several ingredient families, such as colorants, preservatives, and UV filters (Annexes IV, V and VI, respectively) are included [2,3]. It is important to note that although Regulation 1223/2009 came into force in 2013, it is continuously revised and updated (with more than 60 amendments since it came into effect). 

From the point of view of their composition, cosmetics are very complex, with variable matrices formed by a high number of substances from highly lipophilic to moderately polar, or exhibiting basic, acidic, or neutral properties. In addition, it is quite frequent that technical mixtures containing impurities or unknown or banned/unexpected compounds that can be formed by the reaction of compounds become present in the formulation under particular exposition conditions [4,5].

Taking into account that cosmetic products marketed on the European Union must comply with the Regulation [2], and that this compliance must be analytically verifiable, the analysis of cosmetics and personal care products is a challenge. A previous sample pre-treatment before analytical determination is usually required. Sample pre-treatment has two objectives: to ensure the pre-concentration of the target analytes as well as to minimize analysis matrix effects. 

In recent years, several reviews covering this topic have been published [3,5,6,7,8,9,10,11], most of them focused on specific types of cosmetics ingredients such as fragrances, preservatives or dyes since they are the most common compounds present in the formulations [3,9,10]. Regarding the reported sample preparation methodology for cosmetics analysis, traditional liquid–liquid extraction (LLE) and solid–liquid extraction (SLE) are still employed, although the use of advanced techniques such as ultrasound-assisted extraction (UAE), solid phase extraction (SPE), matrix solid-phase dispersion (MSPD) or pressurized liquid extraction (PLE) [3,4,6,7,12] is growing. In recent years, green analytical chemistry (GAC) principles have been increasingly implemented for cosmetics analysis through the miniaturization of classical extraction procedures, as well as the substitution of hazardous chemicals and solvents by environmentally friendly alternatives, as the main objective is to improve their environmental friendliness without compromising method performance [13,14,15].

The separation and determination of the target analytes in the cosmetics matrices are mainly performed by gas chromatography (GC) and liquid chromatography (LC). The choice between them depends on the chemical nature of the target analytes. In general, LC is the preferred option for the most polar compounds such as preservatives or UV filters, especially for hydroxyl preservatives or benzophenone-UV filters derivatives. On the other hand, GC is the preferred chromatographic technique for the separation of volatile or semi-volatile compounds such as fragrances, although non-polar compounds (UV filters, preservatives, etc.) are also successfully analysed by GC. In this case, a derivatization step is included during the sample pre-treatment to modify several chemical functions of the original low-volatile compounds. Derivatization usually employs acetylation with acetic anhydride, or silyl derivatives to improve the chromatographic peak shape of the most polar compounds. This step is usually performed in situ during the extraction step to obtain a good method performance in a short amount of time.

Due to the capability of several compounds present in cosmetics, such as UV filters or some plasticizers, to absorb light in the UV-Vis spectrum, diode-array detector (DAD) was the preferred detection method after LC separation. However, due to its low specificity, which complicates quantifying multiple compounds in complex sample matrices, such as cosmetics, the combination of GC or LC with mass spectrometry (MS) or tandem mass spectrometry (MS/MS) detection became the most suitable option. This approach improves the analytical selectivity and sensibility, allowing the detection of the target analytes at trace levels, which is especially important to identify the impurities and banned compounds. In recent years, the use of high-resolution mass spectrometry (HRMS), mainly based on QTOF detection, has started to play a more important role for multitarget analysis.

**Figure 1:**
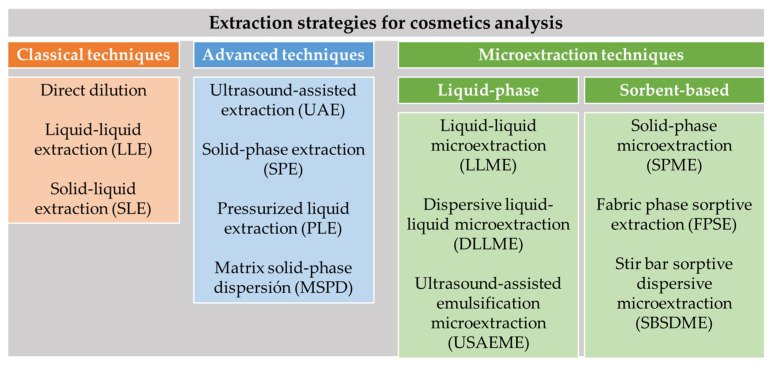
Sample preparation techniques for cosmetics analysis included in this review.

The main objective of this review is to provide an update on the cosmetics and personal care products’ sample preparation methodology. The most relevant analytical methods reported (Scopus database) in the last decade (2010–2020) for the extraction and determination of organic compounds, including cosmetics ingredients such as UV filters, fragrances, preservatives, plasticizers or dyes, as well as the strategies to determine impurities, banned or unexpected compounds (N-nitrosamines, polycyclic aromatic hydrocarbons (PAHs), pesticides, antibiotics, etc.) will be presented. The application of both traditional sample preparation based on direct dilution, LLE or SLE as well as advanced techniques such as ultrasound-assisted extraction (UAE), solid-phase extraction (SPE), pressurized liquid extraction (PLE), matrix solid-phase extraction (MSPD) to cosmetics matrices will be addressed. The recent trends in liquid- and sorbent-based microextraction techniques will be also discussed. Figure 1 illustrates the sample preparation strategies included in this review. The novel developments regarding miniaturization, new sorbent materials with high adsorption properties and the most recent technological trends will also be addressed. 

## 2. Sample Preparation Strategies for Cosmetics Analysis 

Traditional cosmetics sample preparation techniques involve liquid–liquid extraction (LLE) and solid–liquid extraction (SLE). However, the main drawback of SLE and LLE is their high organic solvent (hundreds of mL) and time consumption. On the other hand, modern sample extraction techniques are advancing towards fast sample processing, easy automatization, as well as a reduction in organic solvent volumes, in agreement with the green chemistry principles [13]. In this way, ultrasound-assisted extraction (UAE), solid-phase extraction (SPE), pressurized liquid extraction (PLE) and matrix solid-phase dispersion (MSPD) are techniques that can be considered environmentally friendly, as most of them easily miniaturized [14,16,17]. The most relevant analytical methodologies based on these techniques are summarized in Table 1 and commented on below.

### 2.1. Direct Sample Dilution

Direct dilution (or ready-to-inject) is still employed as a suitable option for simple formulations such as perfumes or liquid cosmetics, but it is not adequate for complex matrices and formulations such as most of the currently marketed cosmetics and personal care products, where a high number of compounds at different concentrations coexist. In addition, from a practical point of view, this approach can negatively affect the chromatographic system. Direct dilution was successfully employed for the determination of phthalates [18,19] and for the simultaneous analysis of fragrance allergens, synthetic musks, phthalates and preservatives [20] from perfumes and simple liquid cosmetic matrices. Ethanol [18,19] or ethyl acetate [20] were the most employed dilution solvents, used in volumes ranging between 1 and 10 mL. For multianalyte determination, the sample:solvent volume ratio was a critical parameter since target compounds can be present at very different concentration levels. In these cases, different dilutions, between 1:10 *v*/*v* and 1:1000 *v*/*v* can be required [20]. Analyses were directly performed by gas chromatography–mass spectrometry (GC-MS) for perfumes, whereas an additional evaporation and reconstitution step was required for the other cosmetics matrices [18]. In all cases, satisfactory performance in terms of recovery (between 80 and 128%) and precision was achieved. 

### 2.2. Liquid–Liquid and Solid–Liquid Extraction (LLE, SLE)

LLE and SLE are two of the oldest sample preparation techniques for cosmetics analysis due to their ease of use and low-cost materials [5,6]. To obtain the highest extraction efficiency in LLE, the extraction solvent should not be miscible with the matrix eluent to solubilize the maximum of analytes. In SLE, extraction is similar to LLE, however, in this case, analytes are dispersed in a solid matrix [21]. SLE and LLE were applied to extract a broad range of organic compounds, including allowed cosmetic ingredients such as preservatives (parabens, isothiazolinones, triclosan, etc.), antioxidants, adipates, UV filters and dyes, as well as banned compounds such as nonylphenols, phthalates, or bisphenol- and dioxane derivatives, among others [22,23,24,25,26,27,28,29,30]. The most critical experimental parameter in LLE and SLE is the extraction solvent. Its selection depends on the target analyte or the analytes’ properties and on the determination technique. Solvents from intermediate polarity (MTBE [22,23,27], acetonitrile [28], mixture of solvents [29]) to more polar ones (MeOH [25,26], EtOH [30], mixture EtOH/water [24]) have been employed. The solvent volume ranged between 1.5 mL and 50 mL and multiple extraction steps were required in some cases to obtain an optimum extraction yield. LLE and SLE procedures were usually assisted by stirring, mechanical shaking, or vortex agitation [22,24,25,27,28,29] to facilitate the analytes’ transfer between the matrix and the solvent. However, one of the main drawbacks of these techniques is the fact that after extraction, further steps such as the centrifugation, concentration and reconstitution of the supernatant or SPE are usually conducted [22,23,24,25,26,27] to obtain clean extracts before analytical determination. 

**Table 1:** Classical and advanced sample preparation strategies for cosmetics analysis: direct dilution, LLE, SLE, UAE, SPE, PLE and, MSPD.

Analytes	Matrix	Sample Amount	Sample Pre-Treatment	Extraction Technique	Extraction/Elution Solvent	Extraction Time (min)	Post-Extraction Treatment	Analysis	Recovery (%)	LOD (ng mL^−1^)	RSD (%)	Ref.
**Direct dilution**												
8 phthalates	Perfumes, shampoos, creams, body milks, shower gels	1 mL (perfumes), 1 g (others)	Dilution in 10 mL H_2_O (other cosmetics)	Direct dilution (perfumes), SLE (others)	10 mL EtOH (perfumes), 10 mL H_2_O + 10 mL MTBE (others samples)		Other cosmetics: centrifugation, drying (Na_2_SO_4_), reconstitution: 1mL EtOH	GC-MS	>80	LOQ: 1000 (perfumes), 200 ng g^−1^ (cosmetics)	<10	[18]
8 phthalates	Perfumes	0.8 mL		Direct dilution	0.8 mL EtOH			GC-MS	88–128	3–294	<6	[19]
Fragrances, synthetic musks, plasticizers, preservatives	Perfumes, colognes	0.1 mL		Direct dilution	Dilution (1:10, 1:100 or 1:1000, *v*/*v*) EtAc			GC-MS	89–110		<6	[20]
**Liquid–Liquid Extraction (LLE), Solid–Liquid Extraction (SLE)**												
Alkylphenols, bisphenols, preservatives	Toothpastes, shampoos, hair conditioners, gels, facial cleansers, hand soaps, sanitary products, lotions, creams, makeup, lipsticks	0.2–0.5 g		SLE	5 mL MTBE	30	Centrifugation, concentration, reconstitution in 3 mL DCM/hexane (1:9, *v*/*v*). SPE, elution: 10 mL DCM/EtAc (1:1, *v*:*v*), concentration	LC-MS/MS	66–135	LOQ: 0.2–1 ng g^−1^		[22]
Bisphenols	Toothpastes, shampoos, face cleansers, bath gels, hand sanitizers, sunscreens, body lotions, lipsticks, hand lotions, hair gels, masks	0.2 g		SLE	5 mL MTBE	20	Concentration, evaporation, reconstitution: 3 mL DCM/hexane (1:9, *v*/*v*). SPE, elution: 10 mL DCM/EtAc, concentration	LC-MS/MS	91–113	1.4–85 ng g^−1^		[23]
4 phenols	Cosmetics			SLE, LLE	EtOH/H_2_O (1:1, *v*/*v*),	0.5 (vortex agitation)	Centrifugation, filtration	LC-MS	90–116	7–15	<8	[24]
8 preservatives, 14 dyes	Lipsticks, lotions, creams, masks, shampoos, gels, soaps, gels, toothpastes	0.1 g	Mixture with 100 mg C18	SLE	1.5 mL MeOH	5 (vortex agitation)	Filtration, dilution MP	LC-MS/MS	70–117	LOQ: 600–3400 ng g^−1^	<16	[25]
6 plasticizers, 7 preservatives	Shower gels	0.1 g		LLE	5 mL MeOH		Dilution in MeOH/H_2_O (75:25, *v*/*v*)	UHPLC-MS	96–105	490–2600 ng g^−1^	<12	[26]
2 preservatives, 2 phthalates	Shampoos, body washes, face cleansers, lipsticks, hair dyes	0.2 g		SLE	5 mL MTBE	30 (shaking)	Centrifugation, evaporation until 1 mL, filtration	LC-MS/MS		5–20 ng g^−1^		[27]
12 preservatives	Creams, gels, lotions	2 g		SLE	25 mL ACN + 25 mL MeOH/H_2_O (1:1, *v*/*v*, 0.6% oxalic acid)	15 (shaking)	Centrifugation, filtration	HPLC-DAD	95–105		<3	[28]
1,4-dioxane	Rinse-off, leave-on cosmetics	0.2 g		LLE, SLE	2 mL hexane/DCM (80:20, *v*/*v*)	3 (vortex agitation)	Centrifugation, SPE	GC-MS/MS	84–108	200 ng g^−1^	<4	[29]
15 UV filters	Sunscreen, creams, balms, after shaves	0.01–0.02 g		SLE	10 mL EtOH		Filtration	HPLC-UV	97–104	30–220	<8	[30]
**Ultrasound-Assisted Extraction (UAE)**												
15 UV filters	Sunscreens	0.1 g	Dilution with 4 mL THF (0.2% ammonium hydroxide), vortex	UAE	6 mL MeOH/H_2_O (80:20, *v*/*v*)	10	Centrifugation, evaporation, reconstitution: 1 mL MeOH, filtration	LC-MS/MS	87–104	2000–20,000 ng g^−1^	<8	[31]
9 preservatives	Lipsticks, foundations, deodorants, lotions, soaps, toothpastes	0.1 g		UAE	5 mL MeOH/ACN (1:1, *v*/*v*),	10	Centrifugation, filtration	LC-MS/MS		900–4200		[32]
Hydroxyethoxyphenyl butanone	Creams, sunscreens, shampoos, soaps, make-up	0.1 g		UAE	10 mL H_2_O		Filtration	LC-UV	86–103	30,000 ng g^−1^	<5	[33]
6 preservatives	Tonics, creams, lotions, shower gels, masks	1 g		UAE	6 mL MeOH	15	Dilution, filtration	LC-DAD	69–119	150–5300	<11	[34]
15 synthetic musks	Perfumes, shampoos, lotions, soaps, antiperspirants, sunscreens	0.03 g		UAE	3 mL (×2) hexane	20	Concentration to 0.2 mL	GC-MS	83–94	0.12–20 ng g^−1^		[35]
ε-aminocaproic acid + amino acids	Cosmetics	0.02 g		UAE (40 KHz, 40 °C)	50 μL NaCl (aq) + 950 μL 0.2 mol L^−1^ borate buffer + 80 μL of 100 mM NBD	10	Dilution, filtration	HPLC-FL	77–122	0.09–0.15	<4	[36]
2 plants derived	Gels, shampoos, creams, antioxidant ampoules, lotions, masks	0.4 g		UAE	25 mL EtOH	1	Filtration, dilution	HPLC-UV	93–115	18–13 μg g^−1^	<9	[37]
4 natural pigments	Temporary tattoos	0.02–0.03 g		UAE (35 kHz)	7.5 mL MeOH	5	Filtration	HPLC-DAD		100–200	<2	[38]
70 compounds: fragrances, preservatives, plasticizers	Masks, baby wipes, extreme cosmetics, borderline products	A wipe/mask, 0.1 g (cosmetics), 0.05 g (products with applicators)	UAE, sup-UAE	UAE (50 KHz, 25 °C)	20 mL (wipes), 2 mL (cosmetics), 5 mL (products with applicators) EtAc	10	Dilution, filtration	GC-MS	70–120		<10	[39]
Free formaldehyde	Hair cosmetics	0.5–1 g	Dilution in 20 mL 2-propanol (0.1%, *w*/*v* SDS), UAE, centrifugation + 2 mL EtOH, dilution	UAE-CPE (50 °C)	6 µmol TB^+^ (0.4 mg L^−1^ sulphite, 0.05% Triton X-114)	15	Dilution to 1.2 mL EtOH	UV-Vis	95	0.38	<5	[40]
2 resin acids	Depilatory wax strips, liquid foundations, mascaras, eyeliner, lip balms	0.05 g		UCSED (60 °C)	500 μL CAN + 200 μL of ANITS solution	45	Filtration	HPLC-DAD, online MS/MS	95–104	8–24	<3	[41]
**Solid-phase extraction (SPE)**												
16 antibiotics	Anti-acne samples	0.5 g	Dilution in 10 mL ACN/H_2_O (2% formic acid, 1:2, *v*/*v*), shaking, UAE, centrifugation, evaporation, reconstitution with 2 mL H_2_O (0.1% formic acid)	SPE (Oasis MCX^®^ 3 cc/60 mg cartridge)	1 mL (×2) MeOH: ammonium hydroxide (4:1, *v*/*v*)		Evaporation, reconstitution: 1 mL MeOH/ACN: H_2_O (5 mM ammonium formate), 12:88, *v*/*v*, filtration	LC-MS/MS	81–113	0.5–1.6 ng g^−1^	<12	[42]
22 coumarin derivatives	Creams, lotions, shampoos, lipsticks	1 g	Dilution in 10 mL MeOH/H_2_O (1:9, *v*/*v*), vortex, UAE, centrifugation, evaporation, reconstitution with 2 mL MeOH/H_2_O (1:9, *v*/*v*)	SPE (Oasis HLB^®^ cartridge)	4 mL MeOH/H_2_O (1:9, *v*/*v*)		Evaporation, reconstitution: 2 mL ACN/H_2_O (70:30, *v*/*v*), filtration	LC-MS/MS	80–93	LOQ: 5–20 ng g^−1^	<15	[43]
NDELA	Soaps, emulsions	0.5 g (water soluble products), 0.1 g (soaps), 0.25 g (water insoluble products)	Dilution in 10 mL H_2_O (water soluble products), 2 mL H_2_O + 8 mL DCM (soaps), 2 mL H_2_O + 4.5 mL H_2_O + 10 chloroform, (water insoluble products), centrifugation	SPE (Bond Elut AccuCAT^®^ cartridge)	1 mL H_2_O			LC-MS/MS	91–114	1	<15	[44]
4 parabens	Creams, make-up, lotions, shampoos, after sun lotions	1 g	Dilution (dil 1:20–1:100) in H_2_O, UAE, centrifugation	SPE (20 mg MWCNTs)	2 mL acetone		Evaporation, reconstitution: 0.5 mL mobile phase	HPLC-C-CAD	82–104	500–2100	<8	[45]
Prednisone	Creams	0.5 g	Dilution in 3 mL NaCl (aq) + 10 mL ACN + 0.2 mL potassium ferrocyanide (aq) + 0.2 mL zinc acetate, UAE, centrifugation	SPE (MIP-MWCNTs)	3 mL (×3) MeOH		Evaporation, reconstitution: 1 mL ACN/H_2_O (40:60, *v*/*v*), filtration	HPLC-UV	83–106	5	<2	[46]
BPA	Shampoos, bath lotions, creams	0.025 g	Dilution in 10 mL NaOH (0.001 M) + 10 mL ethylether, centrifugation + 10 mL toluene	SPE (MIP: imprinted silica NPs)	1 mL MeOH		Evaporation, reconstitution: MeOH/H_2_O (65:35, *v*/*v*)	HPLC-UV/FL	87–97	0.001 micromol L^−1^	<9	[47]
6 benzotriazole UV filters	Lotions, emulsions, creams, sunscreens	1 g	Dilution in 5 mL MeOH, UAE, filtration	SPE (GS cartridge)	0.8 mL acetone			HPLC-UV	89–105	0.03–0.10	<8	[48]
7 sulphonamides	Anti-acne samples	0.2 g	Dilution in 5 mL 0.1 M acetate-ammonium (aq), vortex, UAE	SPE (CGO/PVC cartridge)	2.5 mL MeOH:acetone, (6:5, *v*/*v*)		Evaporation, reconstitution: 0.5 mL MP	IC-UV	88–102	3.4–7.1	<6	[49]
PABA	Creams, sunscreens	0.1 g	Dilution in H_2_O or EtOH	SPE (Ni-Zn-Al(NO_3_^−^)LDH)	2.5 mL NaCl (2M)			UV	96–101	3.7	<4	[50]
9 glucocorticoids	Cosmetics samples	0.2 g	Dilution in 3 mL NaCl (aq), vortex + 2 mL ACN. Centrifugation + 2 mL ACN + 10 mL H_2_O + K_4_[Fe(CN)_6_]·3H_2_O (10%, *w*/*w*) + zinc acetate (20%, *w*/*w*). Centrifugation, filtration	SPE-PMME	0.1 mL ACN/H_2_O (80:20, *v*/*v*, 0.3% formic acid)			LC-MS	84–104	0.1–1.9	<15	[51]
11 N-nitrosamines	Skin care products	1 g	Dilution in 7 mL ACN, vortex, UAE, centrifugation	dSPE-MWCNT (50 mg MWCNT-10)		3	Evaporation, reconstitution: 1 mL MeOH/formic acid (aq, 0.1%) (3:7, *v*/*v*), filtration	LC-MS/MS	89–128	7–250 ng g^−1^	<30	[52]
7 parabens	Creams	0.5 g	Dilution in 1 mL MeOH, stirring, centrifugation. Dilution in H_2_O	VA-d-SPE-MOF (150 mg HKUST-1)	2 mL MeOH	5	Filtration, evaporation, reconstitution: 500 μL of ACN/H_2_O (35:65, *v*/*v*)	HPLC-DAD	64–121	0.1–0.6	<12	[53]
3 preservatives	Sunscreen, antiperspirants, creams, lotions	0.05 g	Dilution in 3 mL MeOH, UAE + 50 mL H_2_O. Filtration + 500 mL with acetate buffer	d-SPE (SBA-15/PANI-*p*-TSA-NCs)	500 μL MeOH (3% *v*/*v* acetic acid)	40		HPLC-UV	82–108	0.1–0.4	<7	[54]
**Pressurized Liquid Extraction (PLE)**												
15 UV filters	Sunscreens, cosmetics	0.1 g	Mixture with Na_2_SO_4_ + 0.6 g sand	PLE (90 °C)	20 mL MeOH/acetone (1:1, *v*/*v*)	10	Dilution in MP	LC-MS/MS	82–101	LOQ: <100 ng g^−1^	<12	[55]
15 UV filters	Sunscreens, hair products, nail polishes, lipsticks	0.1 g	Mixture with 0.6 g Florisil^®^	PLE (90 °C)	10 mL ACN	10	Dilution in EtAc, derivatization	GC-MS/MS	>80		<10	[56]
26 fragrance allergens	Moisturizing creams, lotions, sunscreens, aftersun lotions	1 g	Mixture with 2 g Na_2_SO_4_ + 2 g Florisil^®^	PLE (120 °C)	15 mL hexane/acetone (1:1, *v*/*v*)	15		GC-MS	85–114	12–1800 ng g^−1^	<10	[57]
13 preservatives	Leave-on cosmetics	0.5 g	Mixture with 1 g Na_2_SO_4_ + 1 g Florisil^®^	PLE (120 °C + in situ derivatization)	15 mL EtAc (1:1, *v*/*v*)			GC-MS	74–110	40–1000 ng g^−1^	<10	[58]
PFOS, PFOA	Waterproof sunscreen	4–5 g		PLE (80 °C)	60 mL MeOH	30	Evaporation + 500 mL H_2_O, SPE, evaporation, reconstitution: 0.5 mL MP	LC-MS/MS	67–102		<20	[59]
26 fragrance allergens, 13 preservatives, 15 phthalates, 11 musks	Baby wipes, wet toilet paper for children	Individual wipe		PLE (110 °C)	20 mL MeOH	5	Dilution in EtAc, filtration	GC-MS	75–119	1-31 ng g^−1^	<10	[60]
25 fragrance allergens, 13 preservatives	Baby, child cosmetics	0.5 g	Mixture with 1 g Na_2_SO_4_ + 2 g Florisil^®^	PLE (120 °C)	20 mL hexane/acetone (1:1, *v*/*v*)	15	Derivatization	GC-MS			<12	[61]
**Matrix solid-phase dispersion (MSPD)**												
25 fragrance allergens	Creams, lotions, shampoos, gels, conditioners, soaps	0.5 g		MSPD (2 g Florisil^®^)	5 mL hexane/acetone (1:1, *v*/*v*)		Dilution (1:10, *v*/*v* or 1:1000, *v*/*v*)	GC-MS	75–118	20–600 ng g^−1^	<5	[62]
13 preservatives	Body milks, lipsticks, creams, sunscreens, deodorants, shampoos, soaps	0.5 g		MSPD (2 g Florisil^®^)	5 mL hexane/acetone (1:1, *v*/*v*)		Derivatization, dilution	GC-MS	88–110	1.4–39 ng g^−1^	<4	[63]
4 preservatives	Shampoos, cleansing gels, baby bath gels, creams, make-up	0.5 g		MSPD (2 g Florisil^®^)	5 mL MeOH		Dilution in H_2_O/MeOH (0.1% formic acid, 5 mM ammonium formate (70:30, *v*/*v*))	LC-MS/MS	60–80	LOQ: 6.6–60 ng g^−1^	<11	[64]
18 plasticizers, 12 musks	Shampoos, soaps, body milks, sunscreens, creams, aftershave lotions, deodorants	0.1 g		µ-MSPD (0.4 g Florisil^®^)	1 mL EtAc			GC-MS	84–105	1.4–300 ng g^−1^	<10	[65]
19 dyes	Face paints, make-up, hairsprays, soaps, toothpastes, shampoos	0.1 g		µ-MSPD (0.4 g C18)	2 mL MeOH		Dilution	LC-MS/MS	69–121	17–952 ng g^−1^	<15	[66]
9 dyes	Makeup, lipsticks, toothpastes, creams, shampoos, eye shadows	0.1 g		µ-MSPD (0.4 g Florisil^®^)	2 mL MeOH		Dilution 1:5, *v*/*v* in H_2_O/MeOH, filtration	LC-MS/MS	70–120	0.01–0.62	<15	[67]
63 colorants	69 cosmetics	0.1 g		µ-MSPD (0.4 g sand)	2 mL MeOH		Concentration to 1 mL, filtration	UHPL-Q-orbitrap HRMS	64–128	0.5–100 ng g^−1^	<10	[68]
25 fragrance allergens, 13 preservatives	Shampoos, toothpastes, gels, soaps, sunscreen, lipsticks, deodorants	0.1 g		µ-MSPD (0.4 g Florisil^®^)	1 mL EtAc		Derivatization, dilution	GC, GC-MS/MS	83–115	0.4–37 ng g^−1^ (GC–MS/MS)	<15	[69]
11 preservatives	Liquid cosmetics, gels	0.05 g		µ-MSPD (0.2 g Florisil^®^)	3 mL (×2) EtAc		Dilution	GC-FID	80–124	53–180	<12	[70]
4 preservatives	Skin-, hand-, face creams	0.1 g		µ-MSPD (0.4 g C18)	0.5 mL THF		Dilution in H_2_O, centrifugation	HPLC-UV	63–83	30–40 ng g^−1^	<8	[71]
26 fragrance allergens, 15 plasticizers, 11 synthetic musks, 13 preservatives, 14 UV filters	Sunscreen, hair products, creams, make-up, lip balms, make-up, lipsticks	0.1 g		µ-MSPD (0.4 g Florisil^®^)	1 mL ACN		Dilution, filtration	GC-MS/MS	97–111		<10	[72]
26 fragrance allergens, 13 preservatives, 15 plasticizers, 12 musks	Gels, shampoos, soaps, sunscreen, lotions, body milks, creams, deodorants	0.1 g		µ-MSPD (0.4 g Florisil^®^)	1 mL EtAc		Dilution	GC-MS	80–110	3–700 ng g^−1^	<15	[73]
13 PAHs, 13 pesticides, 8 phthalates, 10 nitrosamines, 2 dyes, 5 fragrances, 6 APEOs	Hand creams, shower gels	0.1 g		µ-MSPD (0.4 g Florisil^®^)	1 mL EtAc (GC-MS), ACN (LC-MS/MS)		Dilution 1:10, *v*/*v* in EtAc (GC–MS) or 1:5, *v*/*v* in ACN/H_2_O; 50:50, *v*/*v*), filtration	GC-MS, LC-MS/MS	72–116	0.09–1.30 ng g^−1^	<15	[74]
2 glucocorticoids	0.2 g		Dilution in 0.3 mL MeOH: H_2_O (1:19, *v*/*v*) + 0.1 g MMIM	MSPD (2 g Florisil^®^)	5 mL MeOH:acetic acid (6:1, *v*/*v*)			HPLC-UV	85–89	200–300 ng g^−1^	<6	[75]

ACN: acetonitrile; C-CAD: corona-charged aerosol detector; CGO/PVC: carboxylated graphene oxide/polyvinyl chloride; CPE: cloud point extraction; DAD: diode array detector; d-SPE: dispersive solid-phase extraction; EtAc: ethyl acetate; EtOH: ethanol; FL: fluorescence detector; GC: gas chromatography; GS: graphene sponge; HPLC: high-performance liquid chromatography; IC: ion chromatography; LDH: layered double hydroxide; LOD: limit of detection; LOQ: limit of quantification; MeOH: methanol; MP: mobile phase; MS: mass spectrometry; MTBE: methyl-tert-butylether; MWCNTs: multi-walled carbon nanotubes; NBD-F: 4- fluoro-7-nitro-2,1,3-benzoxadiazole; RSD: relative standard deviation; SBA-15/PANI-p-TSA-NCs: polyaniline *para*-toluenesulfonic acid nanocomposite supported; SDS: sodium lauryl sulphate; THF: tetrahydrofuran; UCSED: ultrasonic-assisted closed in-syringe extraction and derivatization; UHPLC: ultra-high-performance liquid chromatography; UV: ultraviolet; VA: vortex-assisted.

### 2.3. Ultrasound-Assisted Extraction (UAE)

The fundament of UAE is based on the cavitation phenomena [76]: the creation of small bubbles in the solvent due to the passage of ultrasound waves allowing for a greater penetration of the solvent within the material, thus increasing the surface area. From a GAC point of view, UAE is greener than classical LLE or SLE [77]. The inclusion of ultrasound energy assisting solvent extraction allows the use of less solvents, reducing the extraction time, costs, and improving the extraction yield. 

UAE has been applied to cosmetics to extract UV filters, preservatives, synthetic musks, amino acids, or natural pigments [31,32,33,34,35,36,37,38] from a broad range of matrices including leave-on and rinse-off cosmetics, as well as specific products such as tattoos. The most used solvents for UAE have been water [31,33], MeOH [34,38], EtOH [37], or the combination of different organic solvents [32] or water before LC analysis, whereas hexane and ethyl acetate have been the preferred ones before GC analysis [35,39]. For amino acids analysis, an in situ derivatization was implemented, employing 4-fluoro-7-nitro-2,1,3-benzoxadiazole (NBD-F) [36]. In all cases, quantitative recoveries, and low values for relative standard deviation (RSD) and LODs were obtained.

Additionally, UAE has been successfully proposed for the multianalyte determination of 70 compounds including fragrances, plasticizers and preservatives in baby wipes, masks as well as in extreme cosmetics and borderline products [39]. The possible transfer of plasticizers from the plastic applicators to the cosmetics matrices, and thereby to the consumers, were evaluated, employing a modified version of the classical UAE, called supported-UAE (Sup-UAE). In this approach, a small amount of cosmetic sample (0.05 g) was applied with its corresponding applicator on a small piece (3 × 2.4 cm^2^) of filter paper sheet. Sup-UAE performance was compared with µ-MSPD and classical UAE, obtaining similar results in terms of recovery and precision, demonstrating its suitability, and giving additional information about plasticizers’ migration from cosmetics applicators [39].

The use of UAE combined with cloud-point extraction (CPE) is a simple, low-cost, and sustainable option. It reduces solvent consumption and extraction time, providing high analyte enrichment factors. UAE-CPE has been applied for the extraction of free formaldehyde from hair products [40]. Determination was performed by UV-Vis, obtaining a LOD lower than 0.4 µg L^−1^. The use of ultrasonic-assisted closed in-syringe extraction and derivatization (UCSED) was performed for the determination of two labile resin acids [41]. The procedure included in situ derivatization with 2-(2-(anthracen-10-yl)-1H-naphtho[2,3-d]imidazol-1-yl)ethyl-p-toluenesulfonate (ANITS) and the analysis of HPLC-DAD, and online MS/MS to ensure the confirmation of the target analytes presence in a broad range of cosmetics matrices. In general, UAE performance in terms of accuracy, precision and LODs was similar to that LLE or SLE (see Table 1). However, after UAE, no further clean-up steps before analysis were necessary, reducing the extraction time, whereas after LLE or SLE, a clean-up step, mainly by SPE, is usually implemented [22,23,29].

### 2.4. Solid Phase Extraction (SPE)

SPE is one of the most popular techniques for the extraction of organic compounds from aqueous matrices. SPE allows the isolation of the target analytes from the sample to a solid phase where they are retained. Afterwards, analytes are recovered by elution in a solvent [78]. However, its application to cosmetics analysis usually requires a previous sample pre-treatment, mainly performed by simple dilution in water [42,45,50] or an organic solvent such as MeOH to obtain a liquid phase [43,46]. Undoubtedly, the sorbent selection is critical to obtain a successful SPE method since it plays an important role in the efficiency and analyte selectivity. In recent years, great efforts have been devoted to the development, characterization and application of advanced sorbents to improve analyte selectivity and specificity, with a high sorptive capacity, simultaneously enhancing their physicochemical stability [79,80].

Nowadays, a wide range of existing commercial SPE sorbents are based on classical normal phases (silica, alumina), reversed-phases (C18, C8) and ion exchange sorbents [80].

The commercial reversed-phase water-wettable polymer N-vinylpyrrolidone-DVB sorbent, commonly known as Oasis HLB^®^, presents a hydrophilic–lipophilic balance, thus it can be used for acidic, basic and neutral analytes. Oasis HLB^®^ has been employed to extract coumarin derivatives from cosmetics [43]. Analysis was performed by LC–MS/MS, obtaining recoveries between 80 and 93% and LODs lower than 20 ng g^−1^. Another commercially available mixed-mode cation-exchange reverse phase sorbent, known as Oasis MCX^®^, a sulphonic-acid modified cross-linked polystyrene, has been employed for antibiotics determination in cosmetics [42]. The Bond Elut AccuCAT^®^ cartridge, consisting of a strong cation exchange (SCX), a strong anion exchange (SAX) and C18 was employed for the isolation of the n-nitrosamine NDELA from cosmetics as well as from triethanolamine (TEA), a commonly used cosmetic raw material frequently contaminated with NDELA. This commercial sorbent showed the highest sensitivity and recovery with the least matrix interferences [44]. The selection of the elution solvent is also a critical parameter. It depends on the analyte properties and on the determination technique. Since several of the target compounds are relatively polar, the use of polar solvents such as water [44] or mixtures of water/methanol [42,43] has mainly been employed. In most cases, after SPE, evaporation and reconstitution in the mobile phase composition was carried out to concentrate the extracted analytes before LC-MS/MS analysis [42,43].

#### New Sorbent Materials for SPE

In recent years, new trends in SPE are closely related to the improvement of sorbents that can be more effective to obtain higher analyte enrichment efficiency. These new materials include carbon nanotubes (CNTs), graphene-sponge (GS) and graphene-oxide (GO)-based materials, layered double hydroxides (LDHs) and metal organic frameworks (MOFs)—among others. Their applications as SPE sorbents for cosmetics analysis are presented below.

Carbon nanotubes (CNTs) were introduced by S. Iijima in 1991 [81]. CNTs are cylindrical nanostructures composed of single or multiple rolled graphene sheets. The hollow fibres have a diameter of 1–50 nm with length in the range of microns. They can be classified into single walled carbon nanotubes (SWCNTs) or multi-walled carbon nanotubes (MWCNTs), depending on the number of graphene sheets [82]. MWCNTs have attracted great attention for their specific characteristics to increase sensitivity, improving enrichment and extract clean-up [80]. The combination of SPE with MWCNTs has been proposed to extract parabens from cosmetics prior to corona-charged aerosol detection (C-CAD) [45]. Another advantage of using MWCNTs is the fact that their surface can be functionalized, thus enhancing their sorption performance and selectivity. In this way, the functionalization of MWCNTs with a molecularly imprinted polymer (MIP) is an option that has been employed for the determination of the corticosteroid prednisone in cosmetics [46]. This combination allowed a reduction in the extraction time, and the sorbent material could be reused up to four times without losing extraction efficiency, representing a suitable option for specific analyte extractions in complex matrices. The use of MIPs has also been reported to extract bisphenol A from leave-on and rinse-off cosmetics [47]. The analytical performance was compared with those obtained employing classical C18 sorbents, showing that the use of imprinted silica nanoparticles allowed higher specific extraction, with less interference. Another recently developed nanomaterials employed as SPE sorbent for cosmetics analysis is graphene sponge (GS). This material presents special characteristics such as negligible weight, open-hole structure, high surface area and variable surface chemistry [83]. It has been employed to extract benzotriazole UV filters from water and cosmetics [48]. The sorbent efficiency was compared with the use of MWCNTs and C18. Results revealed that GS showed the best extraction efficiency for the target compounds, which is attributed to hydrogen bonding, π–π stacking and hydrophobic interactions between GS and the benzotriazoles. In addition, the large surface area and pore size of GS probably enhance the extraction capability. Another graphene-based sorbent that exhibits a large surface area, chemical stability and durability is graphene oxide (GO). Its adsorption capacity can be attributed to the delocalized π-electron system, and its properties can be improved by chemicals modifications such as the carboxylation of hydroxyl and epoxy groups on the GO surface, greatly improving its extraction selectivity. In this way, the use of carboxylated GO with polyvinyl chloride (CGO/PVC) was proposed to determine different sulphonamides as contaminants in cosmetics’ products followed by IC-UV analysis [49]. A novel monolithic capillary column with embedded graphene was developed and used for polymer monolith microextraction (PMME). The monolith capillary contained poly butyl methyl acrylate-ethylene dimethylacrylate-graphene (BMA-EDMA-GN) and it was successfully applied for the extraction of nine glucocorticoids from cosmetics. High enrichment capacity was observed in the case of a GN-entrapped monolith showing satisfactory reusability and stability during extraction [51].

Other nano-structured materials gaining special attention in recent years for their special physicochemical properties include layered double hydroxides (LDHs). These are synthetic 2-dimensional nano-structured inorganic materials with general formula: M^2+^_1−x_M^3+^_x_ (OH)_2_]^x+^ [A^n−^_x/n_·mH_2_O]^x−^, where M^2+^ is a divalent metal ion (Zn, Mg, Cu, Co or Ni), M^3+^ is a trivalent metal (Al, Fe or Cr), x is the ratio of M^3+^/(M^2+^ + M^3+^) and *A^n−^* is a *n*-valent anion. A large variety of materials can be obtaining by varying the proportion of the divalent and trivalent cations [84]. The use of a nickel-zinc-aluminium layered double hydroxide (Ni-Zn-Al LDH) was assessed to extract the UV filter p-aminobenzoic acid (PABA) from sunscreens [50]. Results showed quantitative recovery values, a high repeatability and matrix independence even at the low concentration levels showing the suitability of this nano-structured composite.

SPE on-column is the most employed mode [42,43,44,45,46,47,48,49,50], although the on-batch mode has also been reportedly employed. Dispersive (d)-SPE is an alternative approach to conventional SPE. In d-SPE, the sorbent is dispersed into the sample. The close contact between the sorbent particles and the analytes greatly favours the sorption, increasing the efficiency of the whole procedure. Finally, the extracted analytes are eluted from the sorbent [85]. The use of MWCNTs in dispersive (d)-SPE has been proposed to determine volatile-N-nitrosamines in skin care cosmetics [52]. After extraction, the MWCNTs were removed before LC-MS/MS analysis. Analytical performance showed good recoveries and precision, although LODs were quite high compared to those obtained for nitrosamines employing µ-MSPD-GC-MS/MS analysis [74]. The use of nanocomposites as well as metal-organic frameworks (MOFs) is increasing as SPE sorbents in d-SPE applications. MOFs have emerged as new crystalline porous materials with promising applications. They are hybrid inorganic–organic microporous crystalline materials self-assembled straightforwardly from metal ions with organic linkers via coordination bonds. MOFs present interesting characteristics such as a high surface area, uniform structure cavities and a permanent nanoscale porosity, among others. Moreover, the availability of various building blocks of metal ions and organic linkers makes it possible to prepare an infinite number of new MOFs with myriad structures and great potential for diverse applications [86,87]. The efficiency of different MOFs: HKUST-1, MOF-5 and MIL-53(Al) has been assessed to extract parabens from cosmetics [53]. After a deep optimization of the experimental conditions, HKUST-1 proved to be the most suitable sorbent. The whole d-SPE(MOF)-HPLC–DAD showed very low LODs (<0.1 µg L^−1^), and only 0.05 g of cosmetic sample were required. A SBA-15/polyaniline *para*-toluenesulfonic acid nanocomposite supporting d-SPE was developed for the extraction of parabens from cosmetic products. The experimental parameters were optimized by a central composite design. Under the optimal conditions which involve the use of only 10 mg of sorbent, the d-SPE (SBA-15/PANI-*p*-TSA-NCs)-HPLC-UV method was satisfactory validated, showing LODs lower than 0.4 ng mL^−1^ [54].

### 2.5. Pressurized Liquid Extraction (PLE)

PLE is an advanced extraction technique that operates at high temperature and pressure. The elevated temperature enhances the solubility of the analytes, breaking the matrix–analyte interactions, and thus increasing the mass transfer. A clean-up step is frequently performed at the same time as extraction (in-cell clean-up) by adding sorbents at the bottom of the extraction cell [16]. Although it requires solvent volumes similar to those of UAE, PLE provides shorter extraction times, and the currently commercially available instruments present a high degree of automatization.

PLE has been applied to determine UV filters, fragrances, preservatives, perfluorooctanoic acid (PFOA) and its derivates [55,56,57,58,59], and for the simultaneous extraction of musks, preservatives and plasticizers [60,61] in a broad range of cosmetics and personal care products, including those intended for babies and children. The main advantage of PLE is that it drastically reduces the extraction time compared to traditional techniques. For example, only 5 min are needed to extract preservatives, obtaining recoveries ranging from 75 to 119% [60]—whereas UAE or SLE require between 10 and 30 min [28,32]. In addition, depending on the instrumentally available extraction cells, it can be easily miniaturized, reducing the sample size by up to five times and the extraction volume up to two times [55]. Analytical determination after PLE has usually been performed by LC–MS/MS for the most polar compounds such as UV filters or PFOA derivatives [55,59], whereas a derivatization step, mainly acetylation with acetic anhydride and pyridine, was simultaneously performed with the extraction before GC-MS analysis for the most polar target analytes’ determination such as UV filters and preservatives [56,61].

### 2.6. Matrix Solid-Phase Dispersion (MSPD)

In comparison with other extraction techniques that use high pressure such as PLE, or the application of supplementary energy such as UAE, MSPD extraction is performed under ambient conditions, without requiring any special equipment. In addition, the possibility of performing extraction and an in situ clean up would reduce the sample preparation time and decrease the required amount of solvent [88]. MSPD was introduced in 1989 by Barker et al. for the determination of drug residues in animal tissue [89]. Since then, it has attracted growing interest for its versatility and possibility of application to a broad range of solid, semisolid, or viscous matrices. The general procedure consists of the direct mechanical blending of the sample (with a dispersant agent) in a mortar until an homogeneous and dispersed material is obtained, that is then transferred to a cartridge and compressed. Analytes’ elution is performed by passing through the column the correspondent solvent by gravity or vacuum assisted.

MSPD’s first application to cosmetics samples was in 2011 to determine fragrance allergens and multiclass preservatives (parabens, bromine derivatives and antioxidants) in rinse-off and leave-on cosmetics [62,63] as well as in baby care products [61] before GC–MS analysis. The most critical experimental parameters such as dispersant, elution solvent and volume were optimized by DoE (design of experiments) to obtain the highest extraction efficiency. In all cases, the experimental conditions involved the use of 0.5 g of the sample, 2 g of Florisil^®^ as dispersant agent, and elution was accomplished with 5 mL of hexane/acetone (1:1, *v*/*v*). For preservatives’ determination, the MSPD-obtained extracts were derivatized by employing anhydride acetic to improve the chromatographic analysis of those containing hydroxyl groups such as parabens. The acetylation procedure was also optimized to obtain a quantitative reaction yield in a short amount of time (10 min) [63]. Another family of preservatives, isothiazolinones, were also successfully extracted from cosmetic matrices by MSPD. In this case, methanol resulted as the optimal elution solvent, and LC-MS/MS analysis was performed [64]. In all cases, quantitative recoveries and LODs at the low ng g^−1^ were obtained, which were well below the legal requirements for those restricted compounds [2].

**Figure 2:**
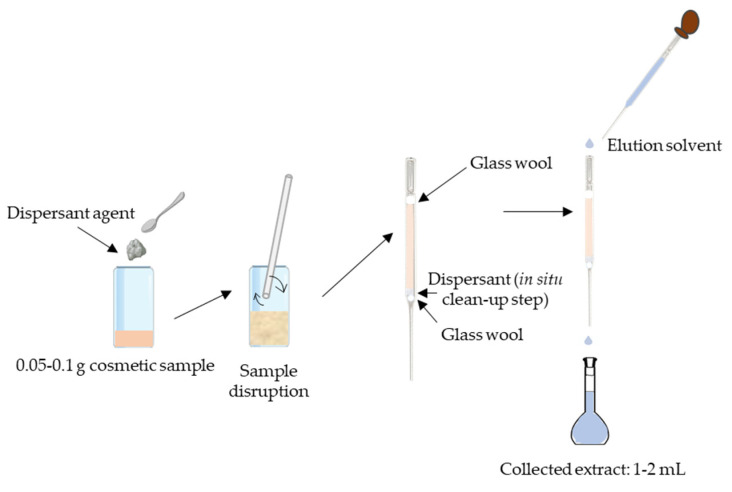
General µ-MSPD procedure employing a glass Pasteur pipette substituting classical cartridges.

In order to reduce the sample and solvent consumption, a miniaturization of the classical MSPD, µ-MSPD, was proposed by Llompart et al. for the first time in 2013 for the simultaneous analysis of plasticizers (phthalates and adipates) and synthetic musks in cosmetics and personal care products [65]. Figure 2 schematically represents the µ-MSPD procedure.

µ-MSPD was subsequently successfully applied for the determination of other cosmetic ingredients including dyes, fragrances, preservatives [66,67,68,69,70,71], as well as for the multianalyte determination of a high number of allowed and restricted ingredients, in addition to banned compounds such as glucocorticoids [72,73,75]. Recently, this miniaturized approach was also applied to extract impurities or unexpected compounds such as polycyclic aromatic hydrocarbons (PAHs), fungicides, nitrosamines, or alkylphenol ethoxylates (APEOs) from cosmetics formulations. In this case, analysis was performed by GC-MS or LC-MS/MS, depending on the chemical properties of the target analytes. In general, obtained LODs were at the very low ng g^−1^ concentration levels, showing that the combination of µ-MSPD with chromatography and mass spectrometry detection is a suitable option to determine the trace levels of impurities and banned compounds with a different chemical nature [74].

It is important to highlight that the use of the µ-MSPD approach allows a reduction in the extraction costs, since it employs disposable common laboratory use material such as pipette tips or glass Pasteur pipettes [65,69,70,72,73,74]. The substitution of the classical plastic MSPD cartridges for glass material is a very suitable and low-cost option for the determination of ubiquitous compounds such as plasticizers, also reducing possible interference during sample preparation [65,73,74]. Other advantages of the µ-MSPD in comparison with classical MSPD is the reduction in the sample amount, reagents, and organic solvents consumption. In most cases, satisfactory results were achieved employing only between 0.05 and 0.1 g of sample [65,67,68,69,70,71,72,73,74] and Florisil^®^ [65,67,70,72,73,74] as the dispersant agent, although the use of other dispersants such as C18 [66] or sand [68] has been reported. Recently, the use of monodisperse molecularly imprinted microspheres (MMIMs) has been successfully proposed to extract glucocorticoids, banned compounds, from cosmetics [75]. The combination of MMIM-MSPD achieved satisfactory recoveries (approximately 90%) and precision (RSD values lower than 6%).

Regarding the elution solvent, ethyl acetate showed the highest extraction efficiency for most of the target compounds before GC analysis [65,69,70,73,74], whereas MeOH was the preferred elution solvent for µ-MSPD extractions before LC analysis [66,67,68]. The use of green solvents such as supramolecular solvents (SUPRASs) has also recently been reported to extract parabens from cosmetics [71], constituting an environmentally friendly alternative to classical organic solvents.

### 2.7. Microextraction Techniques for Cosmetic Analysis

Microextraction techniques involve the use of a small volume of the extraction phase in relation to the sample volume. Although microextraction techniques could not be exhaustive procedures compared with classical ones, they have the advantage of being almost solvent-free and therefore, more sustainable and easily implemented—key factors in current developments for cosmetics analysis [15,90]. The most relevant sample preparation developments for cosmetics analysis based on liquid-phase (liquid-liquid microextraction (LLME), dispersive liquid–liquid microextraction (DLLME), ultrasound-assisted emulsification microextraction (USAEME)) and sorbent-phase (solid-phase microextraction (SPME), fabric-phase sorptive extraction (FPSE), stir bar sorptive dispersive microextraction (SBSDME)) microextraction techniques are shown in Table 2.

#### 2.7.1. Liquid-Phase Microextraction Techniques

Compared with conventional LLE, liquid-phase microextraction (LPME) is faster and more environmentally friendly since a minimal amount—a few microliters—of organic solvents is required. Due to the high ratio of sample volume: extracting solvent, high analytes enrichment can be obtained. In recent years, liquid-based microextraction techniques have become one of the preferred techniques for the extraction of different analytes from various foods and environmental matrices [91]. However, their application for cosmetics analysis may be a challenge due to the high matrix complexity of these products. In this sense, preliminary sample pre-treatments such as sample dilution in organic solvents or water, vortex agitation or UAE are usually required to obtain a liquid phase before LPME. Further steps after extraction are also implemented to concentrate the analytes before analysis [92]. The most employed liquid-based microextraction techniques for cosmetics analysis have been liquid–liquid microextraction (LLME), dispersive-LLME (DLLME) and ultrasound-assisted emulsification microextraction (USAEME). The most relevant methodologies are included in Table 2 and are commented on below.

**Table 2:** Microextraction techniques for cosmetics analysis. Liquid-phase (LLME, DLLME, USAEME) and sorbent-based (SPME, FPSE, SBSDME) methodologies.

Analytes	Matrix	Sample Amount	Sample Pre-Treatment	Extraction Technique	Extraction/Desorption Solvent	Extraction Time (min)	Post-Extraction Treatment	Analysis	Recovery (%)	LOD (ng mL^−1^)	RSD (%)	Ref.
**Liquid-Phase Microextraction Techniques for Cosmetics Analysis**												
**Liquid–liquid Microextraction (LLME)**												
4 alternative preservatives	Creams, gels	0.1 g	Dilution in 5 mL H_2_O, centrifugation	VA-LLME	1 mL hexane	0.3	Evaporation, reconstitution: 400 µL ACN	HPLC-UV	84–118	20–60	<10	[93]
4 preservatives	Cosmetic- oil products		0.5 g	VA-LLME (DES)	200 μL [ChCl-Ethylene glycol (1/2)]			HPLC-UV	>84	50–60	<3	[94]
Bronopol	Creams, shampoos, gels		0.1 g	VAEsME	0.5 mL H_2_O		0.3	HPLC-UV	91–104	900		[95]
**Dispersive Liquid–Liquid Microextraction (DLLME** **)**												
6 preservatives	Aqueous cosmetics	5 g	Dilution in 100 mL H_2_O	DLLME	30 μL chloroform	2		GC-FID	81–103	5–25	<8	[96]
7 preservatives	Lotions, creams	1 g	Dilution in 25 mL H_2_O (lotion), 5 mL EtOH (cream), vortex. UAE, centrifugation	DLLME	0.5 mL isopropyl alcohol		Evaporation, reconstitution: MeOH/H_2_O (20:80, *v*/*v*)	HPCE	71–113	200–375 ng g^−1^	<5	[97]
Acrylamide	Creams, gels	0.15–0.5 g	Dilution in 10 mL H_2_O, vortex + 0.2 g NaCl + 3.5 mL hexane, vortex, centrifugation + 250 μL ethanolic 2-naphthalenethiol (aq) + 250 μL di-sodium tetraborate (aq), MW	DLLME	80 µL chloroform	5	Evaporation, reconstitution: 30 µL EtOH/H_2_O (50:50, *v*/*v*)	LC-UV	85–112	0.7–2.4 ng g^−1^	<14	[98]
Atranol, chloroatranol	Perfumes	1 mL	Dilution in 1.5 mL H_2_O + 1.5 mL hexane, vortex, centrifugation + 8 mL H_2_O + 1 mL K_2_CO_3_ (aq)	DLLME	100 μL chloroform	5		GC-MS	79–110	3-5	<9	[99]
1 preservative	Moisturizer, toner, lotion, soap		Sample dilution (1:100), filtration	VA-DLLME	5 mL chloroform	5		UV-Vis	82–97	476	<6	[100]
4 preservatives	Cream, lotion	0.05 g	Dilution in 200 µL MeOH, vortex, UAE. Dilution in H_2_O (1:100), centrifugation	VA-DLLME	100 µL DES	4		HPLC-DAD		0.3–2		[101]
6 phthalates	Shampoos, gels, creams, deodorants, makeup	0.03 g	Dilution in 3 mL ACN, UAE, centrifugation, filtration	US-DLLME	150 μL CCl_4_	2	Evaporation, reconstitution: 25 μL ACN	HPLC-DAD, LC-MS/MS	84–124	0.04–0.45 (LC-MS/MS)	<10	[102]
5 UV filters	Sunscreens	0.4 mL		US-VA-DLLME	160 μL anisole	5	Evaporation, reconstitution: 20 μL 2-VNT	HPLC-DAD	88–105	15	<3	[103]
6 parabens	Face masks, cream, hair conditioner	0.05 g		UNE-DLLME (35 W)	200 μL octanol	10		GC-FID	82–109	2000–9500 ng g^−1^	<5	[104]
2 dyes	Lipsticks	50 mL		Magnetic stirring assisted-DLLME	500 µL acetone	6		HPLC-DAD	90-95	1	<3	[105]
Vitamin E	Creams, make-up, shampoos	0.5 g	PLE, dilution to 50 mL ACN, filtration	DLLME	100 μL CCl_4_	3	Evaporation, reconstitution: 15 μL MeOH	HPLC-DAD	87–115	3–15 ng g^−1^	<8	[106]
1 dye	Eau de toilette, shampoos		Sample dilution and pH =4 adjustment	IL-DLLME	150 mL of [C10MIM][BF4]		Evaporation, reconstitution: 260 mL MeOH	HPLC-UV	99–103	0.34 ng	<1	[107]
1 preservative		1 g	MSPE, UAE, centrifugation + 100 µL Fe_3_O_4_ NPs suspension + 20 mL H_2_O, vortex	IL-DLLME	100 μL [C_6_MIM][PF_6_]	10		HPLC-DAD	75–98	140	<7	[108]
4 phenolic compounds	Toner, lotion, make-up remover, perfume	1 mL	Dilution in 3 mL ACN, UAE, dilution in 10 mL H_2_O	IL-DLLME	+80 μL [C8MIM] [PF6]	5		CE	82–119	5–100	<13	[109]
6 oestrogens	Lotion		Centrifugation, pH = 4 adjustment, filtration	IL-DLLME	40 mg [P_6,6,6,14_^+^]_2_[CoCl_4_^2−^]	5	Supernatant + 500 μL ACN	LC-UV	96–111	5–15	<10	[110]
4 parabens	Facial tonics		Sample dilution in 100 mL NaCl (aq, 8% *w*/*v*, pH = 5)	IL-DLLME	30 μL C_8_Gu-Cl + 45 μL Li-NTf_2_	5	Microdroplet dilution to 60 μL ACN	HPLC-DAD	82–114	0.5–1.4	<16	[111]
10 phthalates	Emulsions	0.3 g		DLLME-SFO	20 μL 1-dodecanol	10	Ice bath (5 min)	LC-UV			<4	[112]
14 colorants	Lipsticks, eye shadows	0.1 g	Homogenization	MA-DLLME-SFO	1 mL EtOH/H_2_O (pH = 4, 95:5, *v*/*v*)	8	Placing into an ice-bath, filtration	HPLC-DAD	90–106	250–3200 ng g^−1^	<3	[113]
3 alkanolamines, 2 alkylamines	Creams, sunscreen, lotions, shampoos, powders	0.25 g		UA-LDS-DLLME (2.5 mM MSA)	1 mL cyclohexane	15	Filtration	IC-non-suppressed conductivity detection	87–109	72–120	<6	[114]
7 N-nitrosamines	Creams, shower gels	0.1 g	Mixture with 0.2 Na_2_SO_4_ + 5 mL of hexane, centrifugation	RP-DLLME	75 μL H_2_O	0.5		LC–MS	80–113	2–50 ng g^−1^	<10	[115]
NDELA	Creams, shower gels	0.1 g	Dilution in 5 mL toluene, centrifugation	RP-DLLME	125 µL H_2_O	5		HPLC-UV	87–117	1.1	<8	[116]
Free formaldehyde	Gels, masks, creams, shampoos, soaps	0.01–0.1 g	Mixture with 0.4 g MgSO_4_ + 5 mL toluene, centrifugation	RP-DLLME	50 µL H_2_O	5		HPLC-UV	91–113	0.7–2.3	<9	[117]
**Ultrasound-Assisted Emulsification Microextraction (USAEME)**												
5 preservatives	Sunscreen, shampoos, toothpastes	0.1–0.25 g	Dilution in 20 mL MeOH, UAE, centrifugation. Dilution to 50 mL MeOH. Dilution	USAEME	40 µL 1-octanol			HPLC-UV	22–102	0.3–8.3	<10	[118]
9 hormones	Cosmetics		0.2 g	IL-USAEME	125 µL [C7MIM][PF6]	27	Dilution of the IL extraction solution (~100 µL) to 1 mL MeOH	LC	86–109	100–900 ng g-1	<3	[119]
18 fragrance allergens	Eau de toilettes, colognes, perfumes			USAEME-SFOD	50 μL 2-dodecanol	10	Dilution with 20 μL MeOH	HPLC-DAD	90–138	1–154	<12	[120]
5 phthalates	Shampoos, after shave gels, hair sprays	0.03 g	Dilution in 100 μL MeOH + 10 mL H_2_O. Hair spray: Dilution in MeOH	USAEME-SFO	30 μL 1-undecanol	12		HPLC-DAD		0.005–0.01		[121]
5 preservatives	Skin cleansers, toothpastes, creams, sunscreens	0.02 g (skin cleanser), 0.05 g (other)	Dilution in 10 mL H_2_O or MeOH, UAE, filtration. Dilution to 500 mL	UASEME	125 µL 1-octanol + 50 µL tween 20 solution	6		HPLC-UV	70–138	0.03–10	<7	[122]
**Sorbent-Based Microextraction Techniques for Cosmetics Analysis**												
**Solid-Phase Microextraction (SPME)**												
24 fragrance allergens	Balms, creams, deodorants, toothpastes	1 mL		SPME (PDMS/DVB fibre, HS, 40 °C, 20 min)		20		GC-FID	80	7–2700	<6	[123]
16 preservatives	Rinse-off, leave-on cosmetics	0.1 g	Dilution in 10 mL H_2_O + 20% NaCl, *w*/*v* + derivatization	SPME (PDMS/DVB/CAR fibre, DI, 40 °C)		15		GC-MS/MS	>85	6–780 ng g^−1^	<13	[124]
Bronidox	Rinse-off cosmetics	1 mL	Dilution in 10 mL H_2_O + 20% NaCl (aq)	SPME (PDMS/DVB fibre, DI)		30		GC-µECD	70–92	60 ng g^−1^	<10	[125]
11 preservatives	Creams, hair conditioners	0.1 g	UV radiation	SPME (PDMS/DVB fibre, DI)		20	Dilution	GC-MS	54–111	31–170 ng g^−1^	<12	[126]
6 preservatives	Emulsions, lotions, creams	0.02 g		SFE-online-SPME (SFE-CO_2_ (55 °C,), derivatization + SPME (PA fibre, HS, 50 °C)		25		GC-MS	89–172	0.5–8.3 ng g^−1^	<8	[127]
BHT		0.1 g	Dilution in 4 mL H_2_O, vortex, centrifugation	Purge-and-trap-NTD (PDMS/DVB fibre, HS, 60 °C)		30		Portable GC-MS	90–99		<10	[128]
NDELA	Shampoos, body gels, hands soaps	10 mL	Dilution in H_2_O	SPME (Aluminium hydroxide grafted fibre, HS, 70 °C)		15		GC-MS	95–99	1 ng g^−1^	<6	[129]
4 preservatives	Sunscreen, lotions, creams		Sample dilution (1:25, *w*/*v*) in NaCl (aq), UAE	SPME (PEG-DA fibre, DI, 65 °C)		20	Desorption: 1 mL MeOH, filtration	HPLC-DAD	90–98	120–150	<7	[130]
6 PAHs	Cosmetics	0.3 g	Dilution in 50 mL H_2_O	SPME (C_3_N_4_@G fibre, DI, 40 °C)		35		GC-MS	70–118	0.001–0.002	<12	[131]
6 fragrance allergens	Shampoos, creams	0.05–0.1 g	Dilution in 100 mL H_2_O	SPME (β-CD/GO fibre, HS, 70 °C)		40		GC-FID	70–94	0.05–0.15	<11	[132]
MVOCs	Hands cream	0.1 g	Mixture with API^®^ 10S + incubation (37 °C, 24 h)	SPME (PDMS/DVB/CAR fibre, HS, 60 °C)		30		GC-MS				[133]
MVOCs	Shampoos, creams, gels, make-up	0.1 g	Dilution in 2 mL H_2_O + bacterial culture	SPME (PDMS/DVB/CAR fibre, HS, 60 °C)		30		GC-MS				[134]
**Fabric phase sorptive extraction (FPSE)**												
3 preservatives				FPSE (CW-20 coating)	MeOH		Filtration	HPLC-DAD		2.7–3.0	<4	[135]
5 preservatives	Rose waters, deodorants, serums, creams	0.1 g (cream)	Sample dilution in H_2_O, filtration, UAE, centrifugation	FPSE (PEG coating)	0.5 mL MeOH	25	Filtration	HPLC-DAD	88–122	0.2–0.6	<5	[136]
**Stir bar sorptive dispersive microextraction (SBSDME)**												
8 N-nitrosamines	Shower gels, body creams	0.5 g	Dilution in 25 mL NaCl (aq), vortex + 1 mL hexane. Centrifugation	SBSDME (CoFe_2_O_4_/MIL-101(Fe) stir bar)	1 mL acetone	30	Filtration, evaporation, reconstitution: 50 µL H_2_O	LC-MS/MS	96–109	3–13 ng g^−1^	<17	[137]
10 PAHs	Creams, milks, body-milks	4 g	Dilution in 40 mL hexane, vortex, centrifugation	SBSDME (CoFe_2_O_4_-rGO stir bar)	0.5 mL toluene	10	Filtration	GC-MS		0.1–24 ng g^−1^	<10	[138]

2-VNT: 2-vinylnapthalene; API: analytical profile index; CAR: carboxen; CE: capillary electrophoresis; CMA; chlormadinone acetate; DAD: diode array detector; DES: deep eutectic solvent; DI: direct immersion; DVB: divinylbenzene; ECD: electron capture detector; EtAc: ethyl acetate; EtOH: ethanol; FID: flame ionization detector; GC: gas chromatography; HPCE: high-performance capillary electrophoresis; HPLC: high-performance liquid chromatography; HS: headspace; IC: ion chromatography; IL: ionic liquid; LDS: low-density solvent; LOD: limit of detection; LOQ: limit of quantification; MA: microwave-assisted; MeOH: methanol; MS: mass spectrometry; MSPE: magnetic solid-phase extraction; NDELA: N-nitrosodiethanolamine; NPs: nanoparticles; NTD: needle trap device; PA: polyacrylate; PEG: polyethylene glycol; PDMS: polydimethylsiloxane; PLE: pressurized liquid extraction; RP: reversed phase; RSD: relative standard deviation; SFO: solidification of the floating organic; THF: tetrahydrofuran; UAE: ultrasound-assisted extraction; UASEME: ultrasound-assisted surfactant-enhanced emulsification microextraction; UNE: ultrasonic nebulization; US: ultrasonic radiation; UV: ultraviolet; VA: vortex-assisted; VAEsME: vortex-assisted emulsification semi-microextraction.

##### Liquid–Liquid Microextraction (LLME)

LLME was successfully employed to extract alternative preservatives from creams and gels [93]. Extraction was vortex-assisted (VA) to improve its efficiency and a chromophoric in situ derivatization step employing benzoyl chloride was implemented to enhance the analytical response of the target compounds [93]. The combination of deep eutectic solvent (DES) with LLME is one of the novel trends in LPME. DES are considered new and green solvents that can be used in the development of new liquid-based methods. DES-VA-LLME was recently proposed to determine parabens in cosmetics oil products. The employed DES was prepared with choline chloride and ethylenglycol [94]. The whole DES-DLLME-HPLC-UV method showed good recoveries and LODs at the low parts per billion [94]. A variation of VA-LLME, called vortex-assisted emulsification semi-microextraction (VAEsME) was successfully proposed for the determination of the preservative bronopol in cosmetics followed by HPLC-UV [95]. This approach allowed a fast one-step solution-extraction procedure which is very useful for the determination of ingredients with restricted concentrations, such as bronopol.

##### Dispersive Liquid–Liquid Microextraction (DLLME)

DLLME was introduced by Rezaee et al. in 2006 [139]. In DLLME, a small volume of extracting solvent is dispersed by the action of a second solvent into the sample. The abundant contact surface of the fine droplets and the solvent phase greatly enhances the extraction efficiency, thus reducing the solvent volume needed and the extraction time [140]. DLLME has been applied for the cosmetics’ extraction of a broad range of compounds including preservatives, fragrances, phthalates, and acrylamide [96,97,98,99,100,101,102]. The most critical experimental parameters affecting DLLME such as dispersive solvent, extraction solvent and volume, and extraction time, were optimized to obtain the highest extraction efficiency. High density solvents such as chloroform [96,98,99,100], dichloromethane [97] or carbon tetrachloride [102] were the most employed ones, although the use of environmentally friendly alternatives, such as polymeric DES composed of DL-menthol and polyethylene glycol, has been proposed for the extraction of parabens from leave-on cosmetics [101]. Recently, anisole, a bio-derived solvent, was employed for the DLLME of UV filters from sunscreens, showing its suitability as a green alternative to classical organic solvents, not only to determine the target compounds’ concentration in the formulations, but also in the skin after sunscreen application [103]. To improve the dispersion of the solvent into the cosmetic matrix, VA-DLLME has been mainly employed [100,101], although the use of ultrasonic radiation (US), ultrasonic nebulization (UNE) or magnetic-stirring to assist in the DLLME of parabens, phthalates, UV filters and dyes have been also reported [102,103,104,105]. For some analytes, especially for the more polar ones such as atranol and chloroatranol, in situ derivatization was performed to enhance their chromatographic response before GC analysis [99]. The use of DLLME-HPLC-DAD after performing other advanced extraction techniques such as PLE has also been proposed as an effective tool to determine vitamin E in cosmetics [106], showing good recoveries and LODs at the low ng g^−1^.

Other novel approaches in DLLME include methodologies based on ionic liquids (ILs), the solidification of the floating organic droplets (SFOD) or the use of low-density solvents (LDS) to substitute the use of organic solvents. IL-DLLME has been proposed as an environmentally friendly alternative prior to HPLC-DAD or CE analysis to extract dyes, preservatives, phenolic compounds or estrogens from cosmetics [107,108,109,110,111]. Methodologies based on DLLME-SFO were successfully employed for the extraction of phthalates from aqueous cosmetics [112] as well as for dyes from lipsticks [113]. In the last case, the extraction and clean-up steps were integrated to simplify the operation, achieving a high-throughput sample preparation. The main factors affecting the extraction efficiency were optimized by multi-response surface methodology and the validated method was applied to more than 120 cosmetics samples. The performance of DLLME-SFO was compared with LLE, UAE and classical DLLME, showing that DLLME-SFO was the most effective technique to extract phthalates from complex cosmetics matrices [109]. The use of cyclohexane, a low-density and less toxic solvent to disperse the sample (LDS-DLLME) was proposed for the determination of alkanolamines and alkylamines [114]. This approach allows the target analytes to transfer into acidic solutions, while liposoluble substances were dissolved in cyclohexane. The complete extraction was accomplished within 13 min and further purification, or clean-up steps were not necessary.

One decade ago, Hashemi et al. [141] proposed a modification of the original DLLME called reversed-phase DLLME (RP-DLLME), where a small volume of water, used as extraction solvent, is dispersed into a bulk organic solution containing the polar target analytes. RP-DLLME has recently been applied to extract n-nitrosamines [115,116] and free formaldehyde [117] from cosmetics and personal care products before LC-MS [115] or HPLC-UV analysis [116,117]. In all cases, the employed volume of water ranged between 50 and 125 µL, with recoveries and LODs similar to those obtained for the analysis of free formaldehyde by UAE-CPE-UV-Vis [40] or n-nitrosamines employing SPE-LC-MS/MS [44].

##### Ultrasound-Assisted Emulsification Microextraction (USAEME)

USAEME was introduced by Regueiro et al. in 2009 [142]. It relies on the emulsification of the organic extractant in the sample by ultrasonic energy without the need for a disperser solvent, which favours efficiency, achieving the complete extraction of the analytes into the organic phase. This technique shows the advantage of using US to accelerate the mass-transfer process between two immiscible phases, reducing the extraction time. USAEME has been applied to extract preservatives, hormones, fragrances and phthalates from cosmetics matrices [118,119,120,121,122].

The use of ionic liquids (ILs) to substitute the organic solvents (IL-USAEME) has been proposed as an environmentally friendly alternative prior to HPLC-UV to determine three glucocorticoids, one androgen and five progestogens in cosmetics [119]. The combination of USAEME with SFO was applied to determine fragrances and phthalates in both rinse-off and leave-on cosmetic samples [120,121]. In both cases, a few microliters of 2-dodecanol and 1-undecanol were employed, respectively, to perform the extraction. Several parameters influencing the extraction efficiency were optimized. Another environmentally friendly approach involves the use of ultrasound-assisted surfactant-enhanced emulsification microextraction (UASEME), that employs a surfactant and less toxic organic solvent which offers an environmentally friendly procedure. A method based on UASEME-HPLC-UV to determine preservatives in different cosmetic samples was developed. Obtained LODs were similar to those provided by classical USAEME [118].

#### 2.7.2. Sorbent-Based Microextraction Techniques

Although LPME approaches are simple and do not require expensive apparatus, they usually require several steps, and their automation is more complex than that required by sorbent-based microextraction techniques. In addition, the sorbent material can usually be reused, and the procedure implies minimum solvent consumption or even completely eliminating the organic solvents in the whole experimental procedure. Besides, a wider variety of sorbent-based microextraction techniques have been developed in recent years in comparison to LPME, likely due to the versatility of different sorbent materials that can be (i) packed in a small device; (ii) dispersed along the sample matrix, or (iii) coated on a solid support [143]. The most employed sorbent-based microextraction technique for cosmetics analysis has been solid-phase microextraction (SPME); however, in recent years, the application to cosmetics of recently developed sorbent-based microextraction techniques such as fabric phase sorptive extraction (FPSE) or stir bar sorptive dispersive microextraction (SBSDME) is increasing with promising results. The most relevant methodologies are included in Table 2 and are commented on below.

##### Solid-Phase Microextraction (SPME)

SPME was introduced in 1989 by Pawliszyn et al. [144]. Nowadays, it is a well-established green solvent-free extraction technique with a large number of applications in different fields such as food, forensic, biomedical and the environment [145]. One of the main advantages of SPME is that extraction and analytes’ pre-concentration are performed in a single step. In addition, SPME allows the possibility to directly sample the vapour phase in equilibrium with the matrix (headspace (HS) mode), or the matrix extract or solution (direct immersion (DI) mode). Nowadays, a high number of commercially available SPME fibres exist, such as polydimethylsiloxane (PDMS), polyacrylate (PA), polydimethylsiloxane/divinylbenzene (PDMS/DVB), carboxen (CAR) or polydimethylsiloxane/divinylbenzene/carboxen (PDMS/DVB/CAR), among others. The fibre coating selection depends on the target analytes’ properties. Two of the most employed ones for cosmetics analysis were PDMS/DVB and PDMS/DVB/CAR, used to extract fragrance allergens or preservatives [123,124,125], showing good performance in terms of accuracy, precision and low LODs. However, in recent years, trends in SPME for cosmetics are moving towards the development of novel sorbent coatings since commercially available fibre coatings are limited and restrict the wide application of this microextraction technique, especially for multitarget analysis. In this sense, the aluminium hydroxide grafted fused silica SPME fibre was successfully prepared by means of a grafting process for the first time to determine the n-nitrosamine NDELA in cosmetics. The homemade fibre showed good thermal stability, up to 500 °C, as well as a satisfactory repeatability with RSD values lower than 6% [129]. A poly(ethylene glycol) diacrylate (PEG-DA) fibre was proposed to determine parabens in cosmetics products [130]. The PEG-DA polymer coating was covalently attached to the fibre by introducing a surface modification with 3-(trichlorosilyl)propyl methacrylate (TPM). This approach leads to an increase in the surface area, improving the extraction efficiency and showing good repeatability (fibre-to-fibre), although obtained LODs were higher than those provided by commercial fibres [124,127]. The use of single-layer graphitic carbon nitride-modified graphene composite (C_3_N_4_@G), as well as β-cyclodextrin/graphene oxide-modified fibres were employed for the extraction of PAHs and fragrances, respectively [131,132]. In most cases, GC analysis was carried out after SPME [123,124,125,127,128,129,131,132], directly performing analytes’ desorption in the instrument injector—although LC analysis has also been employed [130]. In this last case, the desorption was performed, employing a small volume of organic solvent.

The combination of different extraction techniques with SPME is attracting significant attention. For example, µ-MSPD in combination with SPME was employed as a fast and reliable tool to extract multiclass preservatives (benzoates, parabens, triclosan, butylhydroxy-toluene and butylhydroxyanisole) from cosmetics as well as to monitor their photo-transformation after solar radiation [126]. This is important since cosmetics, especially leave-on ones, are in prolonged contact with the skin, and exposure to solar radiation may not only cause the inactivation of the preservatives, but may also produce potentially hazardous photoproducts. The combination of supercritical fluid extraction (SFE)-online-SPME has been employed to extract multiclass preservatives including parabens and antioxidants from cosmetics [127]. The analytes were extracted from the matrix with supercritical CO_2_. Since analysis was performed by GC-MS, an in situ derivatization step was necessary to improve the chromatographic peak shape of the parabens. Finally, analytes were adsorbed on a polyacrylate (PA) fibre. The combination of a purge-trap with a headspace needle trap device (NTD) and portable GC-MS analysis has also recently been proposed to determine the antioxidant BHT in cosmetics [128], demonstrating the feasibility of utilizing a hand-portable GC-MS for the real-time estimation of this compound, facilitating quality control measurements by inspecting agencies.

SPME has also been proposed as a very promising, non-invasive, and fast technique to evaluate the presence of bacteria (*Escherichia coli*, *Pseudomonas aeruginosa*, or *Proteus mirabilis* among others) in cosmetics. Most of the currently employed techniques for this purpose, including the official methodology, are based on the colony-forming units (CFUs) count and although they employ low-cost materials, they are laborious and require several steps, also being highly time consuming (up to 72 h). It is well known that bacteria produce microbial volatile organic compounds (MVOCs), which are formed during bacterial metabolic processes. This way, SPME employing commercial PDMS/DVB/CAR fibres has been employed to perform an in situ extraction and preconcentration of the MVOCs in a single step [133,134]. Afterwards, GC-MS has been employed as determination technique to unequivocally identify the MVOCs derived from the enzymatic activity, allowing the identification of several of them as specific markers for each one of the studied bacteria, as well as others as general indicators of bacteria presence.

##### Fabric-Phase Sorptive Extraction (FPSE)

Fabric phase sorptive extraction (FPSE) has recently been developed by Furton’s and Kabir’s group [146]. FPSE uses small squares of cellulosic (or other) fabric coated with a thin sol–gel, which are directly immersed in the samples to sorb and extract analytes. Analytes’ desorption is performed by immersing the FPSE device into a small volume of solvent and can be assisted by US or vortex agitation to accelerate the analytes’ transfer to the solvent. The use of a fabric-phase sorptive extraction membrane provides a high surface area which offers high sorbent loading, shortened equilibrium time, and an overall decrease in the sample preparation time. It has mainly been applied to extract different organic contaminants from environmental matrices [147].

Although it is a very recent extraction technique, it has been applied, followed by HPLC-DAD, to the determination of parabens in cosmetics [135,136]. The main experimental parameters affecting extraction such as FPSE membrane coating, sample pH, or desorption solvent were optimized, showing that both carboxen (CW-20) and PEG membrane coatings were suitable to extract parabens. Satisfactory recoveries and precision were achieved and LODs at the ng mL^−1^ were obtained. The FPSE membrane can be reused several times without losing extraction efficiency, showing the suitable applicability of this recent technique to cosmetics.

##### Stir Bar Sorptive Dispersive Microextraction (SBSDME)

Among the sorbent-based microextraction techniques, stir bar sorptive dispersive microextraction (SBSDME) is one of the most recent ones. It was introduced by Benedé et al. as a hybrid microextraction technique that combines the principles of stir bar sorptive extraction (SBSE) and dispersive µ-solid-phase extraction (dµSPE) [148]. It has been mainly applied to determine organic contaminants in the aquatic media [149]. Although it is a very recent technique, its use has been extended to cosmetics matrices using a hybrid magnetic composite made of CoFe_2_O_4_ MNPs and MIL-101(Fe) MOF as a sorbent phase to analyse N-nitrosamines [137] and a magnetic carbonaceous composite made of CoFe_2_O_4_ and reduced graphene oxide (rGO) to extract PAHs [138]. In both cases, satisfactory recoveries and precision were achieved, showing the use of SBSDME as a promising green microextraction technique.

## 3. Future Trends and Directions

In recent years, great efforts have been made to develop robust and reliable analytical methodologies for cosmetics analysis. In this way, sample preparation plays an essential role. Due to the high number of chemical compounds co-existing in these formulations, the development of automated and fast procedures for the simultaneous extraction of a high number of analytes in a single step is necessary. Extraction procedures such as SLE, LLE, UAE, SPE, PLE or MSPD are still in use for cosmetics analysis. However, the trends moving towards miniaturization allow reducing sample, solvent, and time consumption. The improvements made in terms of the miniaturization and portability of extraction devices greatly facilitate the implementation of these techniques in control laboratories and in the performance of in situ analysis. Regarding microextraction techniques, to date, SPME has been the most employed for its simplicity, non-organic solvent use and high analytes’ enrichment. In addition, the variety of commercially available fibre coatings is constantly growing, which are opening up the range for further applications in the cosmetics analysis field. However, the application of recently developed techniques such as FPSE or SBSDME, that have mainly been applied to environmental analysis, in the field of cosmetics is generating growing interest. The development of new sorbent materials such as MWCNTs, LDHs or MIP-based coatings will continue being of particular interest to these approaches. Further directions of research should also consist of developing analytical tools to evaluate the presence of non-expected compounds, such as those of botanical origin, since the presence of cosmetics containing ingredients of natural origin is growing, assessing the stability of these compounds and evaluating the potential by-products formed by photodegradation.

## Data Availability

Not applicable.
